# Awareness, Motivation, and Fear towards Canine Blood Donation—A Survey of Dog Owners in Lithuania

**DOI:** 10.3390/ani11113229

**Published:** 2021-11-12

**Authors:** Brigita Zakarevičiūtė, Dalia Juodžentė, Birutė Karvelienė, Vita Riškevičienė

**Affiliations:** 1 Dr. L. Kriaučeliūnas Small Animals Clinic, Faculty of Veterinary, Veterinary Academy, Lithuanian University of Health Sciences, Tilžės str. 18, 47181 Kaunas, Lithuania; dalia.juodzente@lsmuni.lt (D.J.); birute.karveliene@lsmuni.lt (B.K.); 2 Department of Veterinary Pathobiology, Faculty of Veterinary, Veterinary Academy, Lithuanian University of Health Sciences, Tilžės str. 18, 47181 Kaunas, Lithuania; vita.riskeviciene@lsmuni.lt

**Keywords:** human–dog interaction, canine blood donation, animal welfare, dog, canine, pet, human–animal kinship

## Abstract

**Simple Summary:**

Canine blood transfusions and blood donations are integral to veterinary medicine. Currently, the supply of canine blood products is not meeting the demand, and veterinarians find it difficult to recruit blood donors, especially when animal welfare is a priority. The general aim of our study was to determine how to improve the effectiveness of canine blood donor recruitment. To do this, we conducted a survey to identify the level of dog owners’ awareness about canine blood donation and to help us understand what would motivate people to become a part of the donation process as well as what people are afraid of. The results of our study suggest that donor recruitment could be increased by dispelling the myths about possible complications and by improving communication between veterinary doctors and dog owners, as the awareness of canine blood donation is poor. In conclusion, recruitment strategies should focus on the management of fear regarding canine blood donation, as well as animal welfare and the education of clients, as motivational strategies come second.

**Abstract:**

The recruitment of canine blood donors remains a challenge, especially in countries where blood donation and veterinary medicine are still emerging medical fields. There are few previous studies that have discussed canine blood donation strategies, and the subject of fear and its influencing factors have not been investigated. The main purpose of our study was to investigate dog owners’ awareness, motivation, and fear regarding canine blood donation in order to improve donor recruitment strategies. We created a six-page questionnaire and submitted it to dog owners (*n* = 207) in person. Two-thirds of the respondents (65.7%) were not aware that canine blood donation exists in Lithuania. We did not find any factors that would significantly affect the motivation of respondents toward donation. We found an association between the fear of the owner and the health status of the owned dog (*p* = 0.008), as well as if their animal had needed urgent care in the past (*p* = 0.031). The fact that some participants were blood donors themselves did not affect their motivation, but they were 19.76% less afraid of canine blood donation (*p* = 0.001), as were respondents who were aware of canine blood donation (*p* = 0.004). In conclusion, the recruitment strategy should focus on the management of fear toward canine blood donation and the education of clients, and donor welfare must remain a priority.

## 1. Introduction

Canine blood donation is a noble, altruistic, and empathetic process. It is an act of human–animal kinship and always features three inseparable figures—a veterinarian, an animal owner or caregiver, and a dog.

Numerous techniques such as advanced blood typing [1], crossmatching [2], transfusion collection, administration, blood products production, and complication algorithms have been developed and have improved the safety of blood transfusion in dogs as well as in other pets and livestock over the years [3,4,5,6,7,8]. Blood donor screening by currently available diagnostic tests for blood-borne pathogens minimizes the risks of blood transfusion [9] as well. Blood transfusion is not only the most common practice to save critically ill patients with low blood parameters [3] but it is also a safe procedure if veterinarians follow the necessary protocol [10].

As for canine blood donation, the demand for canine blood components for transfusion has led to the creation of different models for recruiting and maintaining a donor pool and the creation of a number of veterinary blood banks. Scientists state that complication rates for canine blood donation are low [5,11]. For example, when dogs donate blood, a bone marrow regenerative response is induced, which restores depleted blood cells within 10 days after blood donation and maintains the iron status within a calculated reference [12]. Despite the fact that small animal veterinary services are growing, blood services around the world are struggling with a permanent shortage of blood, and the proportion of pets that donate blood is unknown [11].

According to the authors’ observations (at Dr. L. Kriauceliunas’s Small Animal Clinic), anemic dogs that require a blood transfusion are common patients in emergency service. Even though an increase in cases of canine babesiosis in Lithuania was reported more than 17 years ago, in 2004 [13], it still remains the most common cause of blood disorders, followed by rodenticides intoxication, internal bleeding, and other pathologies. Despite the fact that blood products are in high demand, canine blood donation is still an emerging medical field in Lithuania. Unfortunately, the supply of canine blood products does not meet the demand, and veterinarians find it difficult to recruit blood donors. Improving the recruitment and retention of donors remains a high priority in human medicine as well [14,15,16].

There are numerous studies concerning transfusion medicine in dogs and cats, but there are few that discuss blood donation and human–dog interaction. Several previous studies have investigated dog owners’ awareness of and attitudes towards blood donation in the United Kingdom [17,18], but there have been no studies conducted to include the rest of Europe. It is known that socio-demographic factors affect the willingness and motivations of human blood donors [19,20], and therefore, we hypothesize that knowledge and motivation about canine blood donation can vary from human to human and from country to country. As previous studies have investigated neither the fear of canine blood donation nor its influencing factors, we decided to include the analysis of canine blood donation fear factors in our study.

Feelings of satisfaction, greater alertness, and increased well-being are the positive effects elicited by blood donations from human blood donors [21,22]. Among human blood donors, the general reasons/motives for donating blood with the highest ranking of importance are general altruism, social responsibility/obligation, and influence from friends [23]. There are numerous studies that discuss the motivation structures of blood donation in human medicine, but there are few concerning canine blood donation [11,17,24].

Some individuals who do not donate blood themselves present canine blood donation as a chance for them to relieve the feeling of guilt or obligation to society [24]—the authors call it “doing good by proxy”. A study conducted by Wang and Murison [17] revealed that owners were not donating their animal’s blood because they did not know where to take the dog to donate, veterinarians did not express a need for donors, and the owners had a lack of awareness. To improve the effective recruitment of canine blood donors, the understanding of pet owners must be increased as well [18].

The general aim of this study was to identify the fears that deter dog owners from participating in canine blood donation and to determine the motivating factors that would help to develop recruitment strategies for canine blood donors who consider animal welfare, as well as factors that would stimulate the growth of small animal veterinary practices. Through the study, we decided to analyze the factors that affect the fear and motivation of dog owners towards canine blood donation.

## 2. Materials and Methods

### 2.1. Ethical Statement

The study was approved by the Lithuanian University of Health Sciences (LSMU) Bioethics Center (No. BEC-01, 2020). The study was conducted at Dr. L. Kriauceliunas’s Small Animal Clinic at the LSMU (hereafter University Small Animal Clinic) between September 2020 and April 2021. The authors ensured that the data of the study participants were processed and protected in accordance with the laws of the Republic of Lithuania.

### 2.2. Dog Owner Recruitment and Survey Design

A pilot questionnaire in the Lithuanian language was created and pre-tested with 30 LSMU clients to ensure the feasibility of the survey and the readability of the items. After completing the questionnaire, the clients were asked to make written or oral comments on whether the questions were understandable or misleading. The authors evaluated the responses and adjusted the questionnaire.

The questionnaire was submitted by the lead author to the dog owners in person. The dog owners were clients of the University Small Animal Clinic, which is the only veterinary medicine school in Lithuania. All respondents were selected randomly and had to meet the following requirements: older than 18 years, owns a dog, and not a Veterinary Faculty student. The owners were kindly asked to complete the questionnaire while waiting for an appointment in the clinic’s waiting room. As the questionnaire was submitted in person to a veterinary doctor, all respondents were informed about the purpose of the study and assured that the survey was anonymous. The questionnaire was completed by the dog owners anonymously and deposited in a locked box in the reception room by the respondents. All respondents were asked to put a blank questionnaire in the same box if they did not complete the survey for any reason. The dog owners who came for emergency visits or were obviously stressed about the visit were not interviewed out of respect for their well-being and privacy. The box with the questionnaires was emptied every Monday morning by the leading author.

The six-page questionnaire contained 5 parts and 26 items (Likert-type scales and close-ended questions) [25], including the following (Supplementary Materials):

- (A) Information about owned dog(s);
- (B) The level of knowledge about canine blood donation in Lithuania;
- (C) Motivation towards canine blood donation;
- (D) The biggest fears towards canine blood donation;
- (E) Socio-demographic information (gender, age, residential city, education, social status, and a question about the owner being a human blood donor).

In Part B, we first asked if the respondent knew that a dog can be a donor of canine blood. If the person answered “No”, we asked them to continue to Part C; if they answered “Yes”, we asked five more questions. Then, we divided the respondents into two groups—B1 was not aware of canine blood donation (*n* = 136), and B2 was aware of canine blood donation (*n* = 71). We sought to find out whether the participants knew that a blood transfusion procedure is available in Lithuania, what requirements exist for the donors, whether their veterinarian has ever mentioned that their dog is suitable for donation, whether their dog has been a blood donor, and whether they perceive that this procedure seems dangerous to the animal’s life.

To estimate what would motivate dog owners to contribute to the canine blood donation program (Part C), we asked them to mark 12 different statements from 1 to 5 on a Likert-type scale (1—does not motivate, 2—motivates very mildly, 3—motivates mildly, 4—motivates moderately, 5—motivates extremely). After answering all the statements, the minimum possible composite score of motivation was 12 and the maximum was 60.

In order to assess how strongly the interviewees feared canine blood donation and which factors frightened them the most, we presented them with six statements about canine blood donation (e.g., “During the donation, a needle will be inserted into a vein in the neck of the dog and blood will be collected”). On a Likert-type scale, participants rated the statements from 1 to 5 (1—it does not frighten me at all, 2—frightens me very mildly, 3—frightens me mildly, 4—frightens me moderately, 5—frightens me severely). The minimum possible composite score was 6 and the maximum was 30.

The statements in these two sections were formulated in such a way that they could be understood by a person without medical education, and consequently, medical terms were avoided.

The questionnaire was designed with the purpose of investigating the motivations and biggest fears of canine owners towards canine blood donation.

### 2.3. Statistical Analysis

The data were first recorded in Microsoft Excel files, and then, analysis was performed using the IBM SPSS Statistics^®^ software program (Statistical Package for Social Sciences 20 for Windows). The averaged experimental results are expressed as means ± standard deviation (SD), and non-parametric data were processed using the Mann–Whitney test and are expressed as medians and inter-percentile ranges. Data relevant to the participants’ motivating factors and fears were evaluated by the Kruskal–Wallis test and the Chi-square test. The association between the scored Likert-type data of motivating factors, fear, and other survey variables was calculated by Spearman’s rank correlation. A correlation coefficient of 0.9–1.0 was considered very strong, 0.7–0.9 as high, 0.5–0.7 as moderate, 0.3–0.5 as low, and 0.0–0.3 as negligible.

A *p*-value of <0.05 was considered significant.

## 3. Results

### 3.1. Socio-Demographic Information

In total, 250 people were invited to participate in the survey, of which 207 completed the questionnaire and responded to all questions; 13 questionnaires were partially filled out (12 people did not fill out Part E), 21 people refused to fill in the questionnaire as soon as they were invited to participate, and 9 empty questionnaires were found in the box.

Most of the participants were women (65.7%). Almost one-third of the respondents (30.0%) were blood donors themselves, and two-thirds were not (70.0%), but 33.8% were interested in becoming human blood donors. The average age of the participants was 38.70 ± 11.87 years.

Almost half (48.3%) of the respondents were from regions of Lithuania, and 51.7% were from the three largest cities—the capital city Vilnius (20.3%), Kaunas (30.4%), and Klaipėda (1.0%). The participants’ places of residence did not affect the knowledge, the motivating factors, or fear.

### 3.2. Information about Owned Dog(s)

Out of the 207 respondents, three-quarters (75.8%) owned at least one dog and one-quarter owned more than one dog (24.2%) (Table 1).

More than half of the respondents (65.7%) confirmed that their dog or one of their dogs had minor (40.6%) or serious (25.1%) health problems at the time of the survey, and 34.3% said that their animal was healthy. We asked our respondents if they had ever needed emergency care when their (current or former) dog’s life was in danger. More than half of the respondents (60.87%) never needed emergency care, and the rest (39.13%) had needed emergency veterinary service for their dog(s) in the past.

### 3.3. The Level of Knowledge about Canine Blood Donation in Lithuania

Just one-third (group B2) of the respondents were aware that dogs could be blood donors (34.3%), and two-thirds (group B1) were not aware (65.7%). Women (27.5%) were more aware than men (6.8%, *p* = 0.297). Analyzing the responses of group B2 (*n* = 71), we found that 14.1% of the respondents did not know that canine blood donation is available in Lithuania, even if they knew that canine blood donation exists in the world. Only 22 respondents (30.1%) were aware of what requirements apply to canine blood donors and just five (7.0%) were informed by their veterinarian that their dog was suitable for canine blood donation. In group B2, 7.0% of the respondents thought that blood donation is a risky procedure for a dog, 67.6% said it is a safe procedure, and 25.4% did not have an opinion.

Out of the 207 respondents, only five (2.4%) owned dogs who had donated blood in the past. We can conclude that dog owners’ awareness of canine blood donation is poor in Lithuania.

### 3.4. Motivation towards Canine Blood Donation

The median composite score of participants’ motivation was 48.0 (39.50–54.0) (Appendix A).

Whether the participant was a blood donor or had knowledge about canine blood donation did not affect the motivational factors compared to the participants who were not blood donors (*p* = 0.49). There were also no significant differences between the male (48.0 (36.5–53.0)) and female (48.0 (41.0–55.0) composite scores for motivation (*p* = 0.405).

The results of the Kruskal–Wallis test and the Chi-square test of independence revealed that the health status of the owned dog(s) did not affect the motivating factors of the respondent either. The composite score difference between owners whose animals were currently healthy (*n* = 71, 49.00 (42.0–53.0)) and those whose animals had minor or serious health problems (*n* = 136, 48.00 (38.0–55.0)) was only one point and not statistically relevant (*p* = 0.48). We did not find any factors that would significantly affect the motivation of pet owners toward canine blood donation.

### 3.5. The Biggest Fears towards Canine Blood Donation

The median composite score of fear was 16.0 (11.0–21.0). As with motivation, the median composite score of fear was equal for males (16.0 (10.5–21.0)) and females (16.0 (11.0–20.50)) (*p* = 0.702). Participants were most afraid of the fact that complications are possible during donation (3.47 ± 1.36) and least frightened by the fact that, before the donation procedure, the temperature of the dog is measured and its general clinical condition is assessed (1.91 ± 1.44).

The results of the Mann–Whitney and Kruskal–Wallis tests revealed that there was a significant association between the fear of the owner towards blood donation and the health status of their dog. Owners who raised one or more dogs with health problems (median composite score of 18.0 (13.0–22.0)) were more afraid of canine blood donation than those whose dogs were clinically healthy (15.0 (10.0–19.5), *p* = 0.008). A significant association was also found between the single dog owners’ (*n* = 157) fear and the fact that their animal had needed urgent care in the past (*p* = 0.031). Respondents who were blood donors themselves were 19.76% less afraid of the blood donation procedure than those who were not blood donors (Table 2).

Respondents who knew that a dog could be a blood donor and had some knowledge about canine blood donation were 22.22% less afraid of the donation procedure (*p* = 0.004).

We calculated the Spearman correlation coefficient to evaluate the correlation between motivation and fear concerning blood transfusion. There was no significant correlation between motivation and fear of all respondents, but there were some interesting correlations when we grouped the respondents into separate groups. Non-blood donors who were more afraid of canine blood donation were more likely to receive a souvenir if their dog became a canine blood donor (*r* = 0.498, *p* = 0.001).

## 4. Discussion

Canine blood donation is a third-party decision. The individual who is making the donation decision is not the same individual who will undergo the procedure [24]. The owner is the one to make the decision about participating in canine blood donation, but before that, they have to be aware of it.

In contrast to the results attained by Wilder and Humm [18], this study found that pet owners were not aware of canine blood donation but agreed that veterinarians play an important front-line role in improving awareness of canine blood donation. In our survey, only five people said that they were informed by the veterinarian that their dog is suitable for canine blood donation. One of the reasons that people did not donate their dog’s blood was that veterinarians did not express a need for it [17]. We conclude that it is essential for veterinarians to understand what their role is in improving the knowledge of dog owners, as well as what would motivate dog owners to make the decision to participate in canine blood donation and what people are afraid of. In the triad of the veterinarian, the donor, and the owner, the role of the veterinarian is to find a way to attract canine donors, perform the procedures safely, and most importantly, to ensure animal welfare and increase people’s awareness.

When we prepared our survey, in Part C regarding motivation, we listed motivating factors that included moral emotional aspects of the owner and the canine blood donation benefits that would help to assess the health status of the animal and that would save money for the owner as well. We wanted to assess what the intentions of animal owners are and whether or not they interfere with animal welfare. The tension that veterinarians experience in trying to serve the interests of both the animal patients and the paying clients has been called the fundamental question in veterinary ethics [26].

On the one hand, the owner of an animal who voluntarily brings their pet to donate blood deserves gratitude. On the other hand, animal owners can be enticed by gifts, free vaccinations, and blood tests, thus encouraging those people for whom animal welfare is not the priority. Dog blood banks are private and generate revenue, and this can create an environment in which dog owners are lured with gifts, resulting in altruism and empathy being pushed aside.

A chance to save the life of another animal was the most motivating factor in our study. The next two most motivating factors were free annual vaccinations and being given priority for a blood transfusion if needed. It is clear that the two opposite aspects—emotional and beneficial—are parallel. Similar data were also collected by Marantidou [27], revealing the factors that motivate human blood donation in Greece. The majority of respondents (>99.0%) believed that blood donation is an important contribution to other human beings and agreed that incentives such as future blood availability for themselves and their families should exist. A survey of 158 pet owners in the United Kingdom found that beneficence was the most frequent motivation noted by owners willing to let their pets donate blood for both male and female pet owners [18].

As in a previously mentioned study, more women than men agreed to participate in our survey, but the data of our study showed that the composite scores of motivation were the same between men and women. Conversely, in human medicine, most studies found that males are positively associated with donations [28,29], except for a recent study in Hong Kong which concluded that more females than males were blood donors in the young adult population in Hong Kong [30]. A systematic review of 28 articles concluded that perceived health benefits and incentives were stronger motivators for males than females [28].

Our results show that even in the era of social media and self-recognition, the least motivating factor to participate in canine blood donation is a chance to make it public on social networks, along with receiving a souvenir or small gift. Not-for-profit organizations use human donors’ desire to be recognized for their generosity by offering donors branded souvenirs such as stickers, pens, or key rings [31]; however, we see that these days, or in the case of pet donations, this is not so important anymore. These results allow us to conclude that general altruism and social responsibility are above self-seeking intentions, but pet owners expect to receive benefits for their dogs as donors as well.

Researchers claim that avoiding the negative aspects of human blood donation and talking only about the positives discourages potential donors [14]. Godin [32] and Balegh [33] suggest that negative aspects may play an important role in predicting donation intention. Previous studies have described four types of specific fears among existing blood donors, including needles, blood, pain, and fainting. All these fears were associated with lower intentions to donate. Fear related to canine blood donation is associated not with the donor itself but with the owner, and it is as important an aspect as motivation.

Our findings indicate that only one-third of the respondents in Lithuania knew that their dog could be a blood donor. This information is different from that of Wang and Murison [17], who disproved the hypothesis that most owners are not aware that dogs can donate blood. One-quarter of the people who were aware of canine blood donation did not have an opinion on whether blood donation is a risky procedure, and very few respondents (7.0%) thought it is a risky procedure for a dog. Our survey revealed that people who are aware of canine blood donation and those who are blood donors themselves are significantly less afraid of the donation procedure than the rest. We can conclude that awareness is essential for recruiting and attracting dog owners to participate in canine blood donation. Wang and Murison [17] found that one of the most common incentives for donating canine blood in previous non-donors was more information regarding the donation process.

The results from our study addressing fear perception indicate that men and women are equally anxious about canine blood donation. Participants of the survey were most afraid of the complications that donations can cause. DeLuca [11] revealed that canine donation complication rates are comparable to those of human blood donor programs. Acute donor reactions were reported in 2.8%, and after treatment, no lasting adverse effects were reported. We found that people’s motivation was not affected by the health status of their animal, but those whose pets were sick were more afraid of the donation procedure. We may assume that people who have been exposed to veterinary procedures are less likely to participate in canine blood donation.

The chosen methodological approach of this study has limitations. The exact population of dog owners in Lithuania is unknown and the number of participants is small. Even though the survey included people from different regions, it still may not be fully representative. However, we believe these preliminary data may be useful in creating recruitment strategies for canine blood donors in other countries as well. Donor retention is of particular importance for veterinary medicine as well as for human medicine. Repeat donors present an opportunity to save costs associated with recruitment strategies [34]. A longitudinal study following the reasons for continuing blood donation might be useful in this respect.

## 5. Conclusions

The balance between canine blood supply and demand remains fragile, which leads to a constant search for donors. This study suggests that donor recruitment could be increased by dispelling the myths about possible complications during the blood donation procedure and by improving communication between veterinary doctors and pet owners.

We conclude that the general altruism and social responsibility of good owners are stronger motivating factors than self-seeking intentions, but pet owners expect to receive benefits for their dogs as donors as well. Regardless, recruitment strategies should focus not only on people’s motivations but also on the management of fears regarding canine blood donation and the education of clients. Ultimately, motivation and fear management strategies should be used to inspire people to donate their dog’s blood for the first time, but donor welfare must remain a priority.

## Figures and Tables

**Table 1:** Survey respondents’ information about owned dog(s) and level of knowledge about canine blood donation.

Statement	Answer 1	Answer 2
Ownership of dog(s)	Owns one dog (*n* = 157)	Owns more than one dog (*n* = 50)
Current health problems of the own dog(s)	Has minor or serious health problems (*n* = 153)	Does not have any health problems (*n* = 54)
Needed emergency service in the past	Yes (*n* = 126)	No (*n* = 81)
Knows that a dog can be a blood donor	Yes (*n* = 72)	No (*n* = 135)
Owns a dog who donated blood in the past	Yes (*n* = 5)	No (*n* = 202)

**Table 2:** The association between the statements from the survey about fear towards the canine blood donation procedure and the respondent being a human blood donor himself/herself. The values present the means ± standard deviation. Significant *p*-values are highlighted in bold.

Statement	Respondent Is a Blood Donor (*n* = 62)	Respondent Is Not a Blood Donor (*n* = 145)	All Respondents (*n* = 207)	*p*-Value
Before the donation procedure, an intravenous sample will be taken from a limb of your dog.	1.77 ± 1.33	2.38 ± 1.47	2.19 ± 1.45	** 0.003 **
Before the procedure, the temperature of the dog will be measured, and its general clinical condition will be assessed.	1.66 ± 1.28	2.02 ± 1.66	1.91 ± 1.44	0.108
Before the procedure, the dog’s fur will be shaved in the neck area.	2.27 ± 1.51	2.66 ± 1.5	2.54 ± 1.55	0.080
During the procedure, a needle will be inserted into a vein in the neck of the dog and blood will be collected.	2.53 ± 1.39	3.39 ± 1.34	3.13 ± 1.41	** 0.000 **
When collecting blood (about 10 min), the dog will have to sit quietly or lie down on the examination table.	2.69 ± 1.37	3.31 ± 1.34	3.12 ± 1.38	** 0.004 **
Complications are possible during donation (skin irritation, bruising at the blood sampling site, general weakness).	3.03 ± 1.4	3.66 ± 1.29	3.47 ± 1.36	** 0.003 **
Composition score	13.97 ± 6.16	17.41 ± 6.62	16.37 ± 6.66	**0.001**

## Data Availability

The data presented in this study are available upon request from the corresponding author.

## Supplementary Materials

The following are available online at https://www.mdpi.com/article/10.3390/ani11113229/s1.

## Appendix A

**Table A1:** The association between the statements from the survey about motivation towards canine blood donation and the respondent being a human blood donor himself/herself. The values represent the means ± standard deviation.

Statement	All Respondents (*n* = 207)	Respondent Is a Blood Donor (*n* = 62)	Respondent Is Not a Blood Donor (*n* = 145)	*p*-Value
My pet and I would save the life of another animal.	4.51 ± 0.98	4.56 ± 0.78	4.48 ± 0.96	0.49
As a canine blood donor, my dog would be given priority for blood transfusion if needed.	4.25 ± 1.08	3.98 ± 1.31	4.36 ± 0.95	0.49
My dog would get a free vaccination once a year.	4.26 ± 1.16	4.16 ± 1.19	4.30 ± 1.15	0.49
My dog would get blood tests for free.	4.22 ± 1.13	4.05 ± 1.21	4.30 ± 1.09	0.49
My dog would get a free checkup for tick-borne diseases.	4.18 ± 1.15	3.95 ± 1.27	4.28 ± 1.08	0.49
I would do a noble task.	4.17 ± 1.18	4.23 ± 1.137	4.14 ± 1.12	0.49
I could inspire other people for this noble work.	3.89 ± 1.33	3.94 ± 1.34	3.88 ± 1.33	0.49
My dog’s blood type would be determined for free.	3.86 ± 1.33	3.69 ± 1.41	3.93 ± 1.29	0.49
I could be proud of my pet.	3.75 ± 1.44	3.81 ± 1.41	3.73 ± 1.45	0.49
I will be able to tell other people about this noble work.	3.39 ± 1.52	3.60 ± 1.42	3.30 ± 1.56	0.49
My dog would receive a souvenir showing that they were a participant in a canine donation program.	2.95 ± 1.56	2.76 ± 1.53	3.03 ± 1.56	0.49
I could make this noble work public on social networks.	2.87 ± 1.65	3.00 ± 1.71	2.81 ± 1.62	0.49
Composition score	46.29 ± 10.35	45.73 ± 11.28	46.53 ± 9.95	0.49
Composition score ^1^	48.0 (39.50–54.0)	47.0 (38.0–55.0)	48.0 (40–54.0)	0.49

^1^ Composition score represented by median level and percentiles.
