# Supplementary material for: Awareness, Motivation, and Fear towards Canine Blood Donation—A Survey of Dog Owners in Lithuania
Source: Animals (Basel). 2021 Nov 12;11(11):3229. doi: 10.3390/ani11113229 (PMC8614293; doi:10.3390/ani11113229)
Supplement: Supplementary file 1 [file animals-11-03229-s001.zip › animals-1411340-supplementary.pdf]

SURVEY OF DOG OWNERS ABOUT DOG BLOOD DONATION

Good afternoon,

I am Brigita Zakarevičiūtė, a PhD student at the Veterinary Academy of the Lithuanian University of Health Sciences and a veterinary surgeon. I am currently conducting research on the topic of dog blood donation, so I would like to know your opinion on this topic. This questionnaire is anonymous.

If you have further questions, you can apply - brigita.zakareviciute@lsmuni.lt

PART (A). In this section, I would like to ask questions about your dog(s). Please mark the correct answer or write down the answer.

A1. How many dogs do you own?

- one
- more then one (***continue from question A7***)

A2. What is the breed of your dog? (if you own a mixed breed, please write it down as well)

- ______________________

A3. What is the age of your dog?

- ______________________

A4. What gender is your dog?

- female
- male

A5. Does your dog currently have health problems?

- no
- yes, have minor health problems
- yes, have serious health problems

A6. Did your dog have ever (currently bred or formerly) needed urgent veterinary care when the life of the animal was in danger? (***please continue from Part B)***

- yes
- no

A7. How many dogs do you have?

- ______________

A8. What are the breeds? (if you own a mixed breed, please write it down as well)

- ____

A9. What is the age of you dogs (please write down the interval from the youngest to the oldest):

- ______________

A10. What are the genders of your dogs?

- females
- males
- females, males

A11. Does any of your dog currently have health problems?

- no
- yes, have minor health problems
- yes, have serious health problems

A12. Did any of your dogs have ever (currently bred or formerly) needed urgent veterinary care when the life of the animal was in danger?

- yes
- no

PART (B). In this section, I would like to ask you what do you know about dog blood donation in Lithuania. Please mark the correct answer.

B1. Do you know that dog can be a dog blood donor?
- o yes
- o no *(please continue from Part C)*

B2. Have you ever heard (knew) that blood transfusions are performed in dogs in Lithuania?
- o yes
- o no

B3. Do you know what requirement apply for dogs blood donors?
- o yes
- o no

B4. Has your dog been a blood donor?
- o yes
- o no

B5. Has your dog's veterinarian mentioned to you before that your dog is eligible to become a blood donor?
- o yes
- o no
- o I cannot remember

B6. Do you think collecting donor blood from a dog it is a risky procedure for a dog’s health?
- o yes
- o no
- o I don’t have an opinion

PART (C). In this section, I would like to find out what would motivate you, as a dog owner, to participate in a dog blood donation program. Please mark the number from **1 (“doesn’t’ motivate me at all“) to 5 (“motivates me a lot“).**

I would do a noble task.	1	2	3	4	5
My pet and I would save the life of another animal.	1	2	3	4	5
As a canine blood donor, my dog would be given priority for blood transfusion if needed.	1	2	3	4	5
My dog would get blood tests for free.	1	2	3	4	5
My dog’s blood type would be determined for free.	1	2	3	4	5
My dog would get a free check up for tick-borne diseases.	1	2	3	4	5
My dog would get a free vaccination once a year.	1	2	3	4	5
My dog would receive a souvenir showing that they were a participant in a canine donation program.	1	2	3	4	5
I could be proud of my pet.	1	2	3	4	5
I will be able to tell other people about this noble work.	1	2	3	4	5
I could make this noble work public on social networks.	1	2	3	4	5
I could inspire other people for this noble work.	1	2	3	4	5

PART (D) If your dog would fit the requirements to become a blood donor, how worried or scared would you be about these statements? Please mark the number from
1 (“doesn’t frighten me at all“) to 5 (“it frightens me a lot“).

Before the donation procedure, the intravenous sample will be taken from a limb of your dog.	1	2	3	4	5
Before the procedure, the temperature of the dog will be measured, and its general clinical condition will be assessed.	1	2	3	4	5
Before the procedure, the dog's fur will be shaved in the neck area.	1	2	3	4	5
During the procedure, a needle will be inserted into a vein in the neck of the dog and blood will be collected.	1	2	3	4	5
When collecting blood (about 10 min) the dog will have to sit quietly or lie down on the examination table.	1	2	3	4	5
Complications are possible during donation (skin irritation, bruising at the blood sampling site, general weakness).	1	2	3	4	5

PART (E). In this section, I would like to ask information about you. I remind you that the questionnaire is anonymous and your personal data will not become public or available to anyone - it will only be used to summarize the purposes of this investigation. Please mark the correct answer or write down the answer.

E1. What is your gender?
- female
- male

E2. What is your age?:
- ______ y.

E3. Where do you live?:
- __________________

E4. What is your educational degree?
- some high school, no diploma
- high school graduate
- professional degree
- bachelor's degree
- master's degree
- other

E5. What is your current social status? (you can mark more then one)
- high school student
- student
- worker
- unemployed
- retired
- other

E6. Are you a blood donor by yourself?
- yes
- no, but I would like to
- no, I wouldn't like that

Thank you for your time and your asnwers.
